# Organizational support and job crafting with the new math teachers’ well-being: The mediating effect of basic psychological needs

**DOI:** 10.3389/fpsyg.2022.961702

**Published:** 2022-11-03

**Authors:** Limei Wang, Fuqiang Peng, Naiqing Song

**Affiliations:** ^1^School of Mathematics and Statistics, Southwest University, Chongqing, China; ^2^School of Science, West Yunnan University, Lincang, Yunnan, China; ^3^School of Foreign Languages, West Yunnan University, Lincang, Yunnan, China; ^4^Centre for Collaborative Innovation of Assessment Toward Basic Education Quality, Southwest University, Chongqing, China

**Keywords:** mathematics teachers, occupational well-being, structural equation modeling, organizational support, job crafting, basic psychological needs

## Abstract

Enhancement of the teacher well-being level has grown into a general, pressing problem to be solved in the domain of education worldwide. Based on the theoretical perspective of the multi-level dynamically formed mechanical model of occupational well-being, this study initially constructed a mechanism model with the occupational well-being role of organizational support, job crafting, and the occupational well-being of new math teachers at primary and junior high schools, and conducted empirical research using structural equation modeling. The study found out that organizational support, job crafting and basic psychological needs have prominent and positive effects on the occupational well-being of the new math teachers in primary up to junior high schools. It also argues that basic psychological needs may mediate the correlation between organizational support, job crafting, and the occupational well-being of the new math teachers. To sum up, the study findings reveal the mechanisms of the role in organizational support and job crafting on the occupational well-being of new primary up to junior high school math teachers. Also, the findings may be conducive to extending the research on the factors that influence the teacher well-being, notably fostering the study on that in the math teachers in the primary up to junior high schools of China.

## Introduction

Well-being is an ideal state of people’s psychological functioning and experience (Ryan and Deci, 2001). Teacher well-being is a response of teachers to their occupation and career from the cognitive, emotional, health, and social aspects (Organization for Economic Co-operation and Development [OECD], 2020), as well as the driving force for development and progress of the teaching community and educational organizations as large (Li and Lu, 2020). The well-being of teachers by profession is first included as a crucial part of the PISA2021 math framework in the OECD report, Teachers’ Well-being: A Framework for Data Collection and Analysis (Organization for Economic Co-operation and Development [OECD], 2020). China (2018) has also promulgated the Opinions of the State Council of the Central Committee of the Communist Party of China on Comprehensively Deepening the Reform with the Teachers in the New Era. The country also proposes that “by 2035, the teachers nationwide will enjoy the well-being at the teaching positions, achievements in the cause, and honors amid the society, as an admirable profession”. Although there are more countries realizing the essential value of the teacher well-being (Selwyn and Riley, 2015; McCallum et al., 2017; AsiaSociety, 2018; UnitedKingdom, 2018), the teacher well-being at primary and junior high schools generally remains low (Schleicher, 2018) and has led to high teacher-drain rate (Collie and Martin, 2017), serious teacher-shortage (Schleicher, 2018) and little teacher-attractiveness (UnitedKingdom, 2018) at the schools. Relevant statistics indicates that approximately 30% of primary and junior high school teachers in the United States quit within 5 years after their university graduation and the teaching job placement (McCallum et al., 2017); 13 European Union countries are already facing a severe shortage of primary and junior high school teachers (Organization for Economic Co-operation and Development [OECD], 2020); and less than 10% of primary and junior high school teachers in France, Spain and Sweden retain their teaching profession as meritorious and meaningful (Organization for Economic Co-operation and Development [OECD], 2019). These figures showcase an increasingly serious problem of low occupational well-being for primary and junior high school teachers (Schleicher, 2018), and raising the teacher well-being has grown into a common, urgent issue in the educational domain worldwide (Aldrup et al., 2018; Braun et al., 2019; Holzberger et al., 2021). Therefore, it is of great pragmatic significance to explore in depth the influencing factors and their role mechanisms with the occupational well-being of primary and junior high school teachers.

A review of the existing literature reveals that researchers also analyze the factors influencing the teacher well-being from different perspectives. In his study, Warr (1994) finds in his study that the teacher well-being may be affected by age and teaching experience and illustrates a U-shaped curve relevant to the two factors. Mattern and Bauer (2014) also see that rational self-planning could enhance the teacher well-being conquest to some extent. Moè (2016b) confirmed that harmonious passion and teachers enthusiastic, have been found to relate with teacher well-being (Burić and Moè, 2020). It has been proved that when teachers experience wellbeing they are also more motivated (Klusmann et al., 2008; Moè, 2016a; Shoshani and Eldor, 2016). Positive organizational psychology is becoming a heated topic in the study of factors influencing the occupational well-being of primary and junior high school teachers (Schleicher, 2018). Organizational support has received increasing attention as a critical driver of the teacher well-being (Lauermann and König, 2016), promoting the teacher identity, forming positive perceptions and eventually, enhancing the teacher well-being (Wang and Xu, 2008). Different perception levels of the organizational support are related to whether teachers can achieve their psychological satisfaction, which further affects the teacher well-being (Wang and Xu, 2008). Some other researchers focus on the positive role of the organizational support perception in job crafting (Kim et al., 2018). This is believed to have broken through the traditional top-down model designed by the school management for primary and junior high school teachers. Job crafting may enhance their sense of belonging and identity at the schools and in turn, heighten their occupational well-being (Wei et al., 2018). In addition, some researchers discovered that the higher the level of satisfaction of an individual’s basic psychological needs is, the more happiness he or she will experience (Wu et al., 2018), and teacher psychological need satisfaction is considered a possible mediator (Moè and Katz, 2020). Teachers’ need satisfaction is linked with reappraisal, which is related to the autonomy supportive (Moè and Katz, 2021). In recent years, in attempt to explore the multivariate interaction factors affecting the occupational well-being of primary and junior high school teachers, some researchers have observed a significant, positive correlation between the sense of organizational support plus job crafting and the subjective occupational success in the school teachers (Zhao and Li, 2019), of which, occupational well-being is deemed to be the main expression form of subjective occupational success (Spurk et al., 2019). Based on the aforesaid studies on the well-being of primary and junior high school teachers, researchers would combine organizational support with job crafting. Then, how do organizational support and job crafting affect the occupational well-being of primary and junior high school teachers? What is the role mechanism of basic psychological needs in organizational support, job crafting, and teachers’ perceptions of their occupational well-being? It is worthwhile to explore these questions in depth.

Literature review as mentioned earlier facilitates applicable exploration for further study on the teacher well-being at primary and junior high schools. However, the existing studies have not yet fully explained the above issues and still leave room for the following study areas.

Firstly, there is a disconnect with each other between the study on organizational support’s facilitation of the teacher well-being and that on the job crafting for the same purpose, with a lack of study on the relationship among the studies on organizational support, job crafting, basic psychological needs, and the teacher well-being, as well as their specific role mechanisms. Some researchers argue that basic psychological needs is a fundamental entry point for the study on the correlation between job crafting and the teacher well-being (Wang et al., 2020), there are still very few studies that introduce basic psychological needs as an intermediate variable to the study on the correlation among organizational support, job crafting, and the teacher well-being.

Secondly, Chinese primary and junior high school teachers also face low levels of the teacher well-being, for which some researchers have analyzed the factors influencing the teacher well-being (Zeng et al., 2021). However, still there is a lack of empirical study on the well-being in the Chinese context.

Thirdly, relevant statistics show that new teachers have relatively low occupational well-being (McCallum and Price, 2010). However, few studies on the teacher well-being have been conducted so far with new teachers at primary and junior high schools. Moreover, there are even much fewer studies focusing on the particular group of teachers of a specific subject at primary and junior high schools (McCallum et al., 2017). Besides, some researchers have also noticed the relatively low happiness of math teachers (Organization for Economic Co-operation and Development [OECD], 2020). Therefore, the study on the well-being of new math teachers is very important.

The contributions of this manuscript are presented in four aspects. First, the multi-level dynamic formation mechanism model of occupational well-being suggests that organizational practices and characteristics have a direct positive influence on occupational well-being, indirectly affecting the well-being by person-environment fit and achieving the well-being through the satisfaction of basic psychological needs. This study intends to explore the correlation between organizational support, job crafting, and the teacher well-being from a multi-level dynamic formation mechanism model of occupational well-being. Second, this study also proposes the hypothesis that basic psychological needs has an intermediary effect on promotion of the teacher well-being via organizational support and job crafting. Third, by constructing a role mechanism model of organizational support, job crafting and the teacher well-being, an empirical study has been conducted using questionnaire data of new math teachers at primary and junior high schools in China. Fourth, the study expands the scope of the study on the teacher well-being from a positive organizational behavioral perspective, opening up new ideas for the government and schools’ building and managing the teaching force, and furthering the professional teacher development.

The manuscript is organized as follows. “Literature review and hypotheses development” section presents the theoretical background and review the literature to develop our research hypothesis tested in this study. Next section presents the sample and methodology used to test the hypothesis. “Results” section shows the empirical results, with “Discussion” section discussing these results. Finally, conclusions are made in section “Conclusion”.

## Literature review and hypotheses development

### The organizational support and the teacher well-being

Organizational support is the extent to which an organization values the contribution of its employees and cares about their well-being (Rhoades and Eisenberger, 2002), as well as the overall perception of employees whose work is recognized and whose well-being administered by the employers (Eisenberger and Huntington, 1986). The school teachers’ perceptive levels of organizational support are related to a teacher’s willingness to work together in behavioral solidarity and to their psychological satisfaction (Eisenberger and Huntington, 1986). Teachers tend to be more satisfied with their profession when they feel supported by their institutions (Tan et al., 2007). And with job satisfaction as a critical factor in well-being, it is clear that increased job satisfaction can significantly enhance occupational well-being (Hur et al., 2016). In addition, some researchers have also found that organizational support to primary and junior high school teachers is remarkably and positively related to various dimensions of well-being, with a significant, positive impact on the teacher well-being (Wang and Xu, 2008). Recently, organizational support has been the strongest predictor of the teacher well-being, enhancing or reducing the effectiveness in terms of psychological capital and emotional exhaustion (Wang et al., 2020). Based on this, the following hypothesis is formulated for this study:

H1: Organizational support have a remarkably positive effect on the occupational well-being of new math teachers at primary and junior high schools.

### The job crafting and the teacher well-being

The essence of job crafting lies in that “employees, on their own initiative, would make bottom-up changes in the content and methods of their work to meet their own needs or those of their groups, for a sense of meaningfulness at work” (Bolino and Grant, 2016; Akkermans and Tims, 2017). Job crafting may help improve the fit between employees and their work environment, enabling the employees to realize their self-worth and create more value for their workplace, thus enhancing their well-being at work (Zhang and Parker, 2019). Furthermore, researchers argue that *t* job crafting may impact positively job satisfaction (Slemp and Vella-Brodrick, 2013; Kim et al., 2018), a significant expression of occupational well-being. Likewise, Wei et al. (2018) argue that teachers’ own competence in job crafting would be a key factor to enhance the teacher well-being. Besides, it has also been suggested that there is a positive correlation between job crafting and the employee well-being, and that employees’ perceptions of well-being are significantly enhanced from their proactive job redesigning behaviors (Hou et al., 2021). Based on this, the following hypothesis is proposed in this study.

H2: There was a significant, positive effect of job crafting on the occupational well-being of new math teachers at primary and junior high schools.

### The mediating effect of basic psychological needs

Basic psychological needs is derived from the self-determination theory proposed by Deci and Ryan (2012), which states that when people’s needs for autonomy are met, they tend to be more actively engaged and creative in their daily activities and be observed to exhibit the individual well-being (Deci and Ryan, 2000). The positive correlation between basic psychological needs and well-being has been affirmed by several studies (Patrick et al., 2007; Wang et al., 2015), which also conclude: the higher level of satisfaction of basic psychological needs in individuals, the greater well-being one may experience (Wu et al., 2018). Within the theoretical framework for the person-environment fit, the self-determination theory expounds the psychological mechanisms by which the fit may affect the well-being at work, and confirms the intermediary role of human psychological need satisfaction (Greguras and Diefendorff, 2009; Yen, 2012; Slemp and Vella-Brodrick, 2013).

Furthermore, organizational support helps improve basic psychological needs (Deci et al., 2016). Also, organizational characteristics (e.g., organizational support) positively or indirectly impact occupational well-being through the person-environment fit resulting from the satisfaction of basic psychological needs (Zou et al., 2015). In addition, some researchers have argued that employees’ job crafting have a positive impact on their basic psychological needs (Van Wingerden et al., 2017). Therefore, job crafting can enable employees to meet their psychological needs and increase their perception of happiness. That is, the basic psychological needs are intermediary between job crafting and employee well-being (Hou et al., 2021). Therefore, the study intends to apply the basic psychological requirements of primary and junior high school teachers to the school environment (Huang, 2021), for which the study also develops the following hypotheses:

H3: Basic psychological needs has a significant positive effect on the occupational well-being of new math teachers at primary and junior high schools.

H4a: Organizational support has a significant, positive effect on basic psychological needs.

H4b: Basic psychological needs have a mediating effect in the correlation between organizational support and the occupational well-being of new math teachers at primary and junior high schools.

H5a: Job crafting has a significant, positive effect on basic psychological needs.

H5b: The basic psychological needs have a mediating effect in correlation between job crafting and the occupational well-being of new math teachers at primary and junior high schools.

**FIGURE 1:**
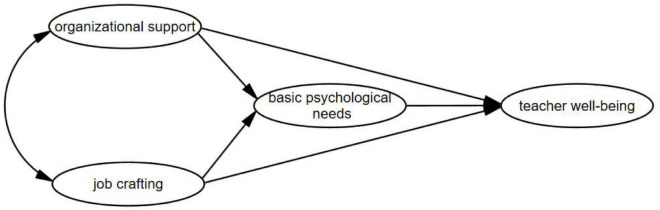
The theoretical model.

In conclusion, this study construct the theoretical model (see Figure 1).

## Methodology

### Sample and data collection

In this study, we selected new math teachers at primary and junior high schools as the subjects. Then we conducted an online questionnaire survey in which new teachers referred to those who had worked for no more than 3 years, according to the existing literature (Huang, 2021). Before the actual study, the researcher conducted a pre-survey with some new teachers in Yunnan province of China. We revised the questions repeatedly to constitute the formal questionnaire. We mainly took a wide range of samples from the provinces of Yunnan, Guizhou, Guangxi, Guangdong, and Shandong, with a total of 280 questionnaires distributed and 262 returned. As a result, 248 questionnaires were deemed valid, excluding incomplete and invalid ones, with a correct return rate of 88.57%. The results of the demographic analysis of the samples are shown in Table 1. We took all the samples from primary and junior high school math teachers who had just worked for 3 years or less. 34.8% of the teachers were males and 65.2% females, which conformed with the essential criteria for this study.

**TABLE 1:** Composition of samples (*N* = 248).

Category	Indicator	Time-frequency	Rate-frequency	Category	Indicator	Time-frequency	Rate-frequeny
Gender	Male	87	35.1	Age	Aged 20 or	1	0.4
	Female	161	64.9		Aged 21–23	66	26.6
Teaching experience	≤1 Year	119	48		Aged 24–26	92	37.1
	1–2 Years (excl. 2 Y)	28	11.3		Aged 27–30	23	9.3
	2–3 Years (excl. 3 Y)	16	6.5		Aged 30 or above	66	26.6
	3 Years	85	34.3	Academic qualification	Under junior college	1	0.4
Professional title	Not conferred	145	58.5		Junior college	24	9.7
	Level-III	30	12.1		Undergraduate	222	89.5
	Level-II	41	16.5		Postgraduate	1	0.4
	Level-I	32	12.9	Teaching level	Primary school	168	67.7
School location	County/district/city	61	24.6		Junior high school	80	32.3
	Village/town (ship)	187	75.4				

### Variables and measurement

To ensure the reliability and validity of the measurement instrument, this study intends to apply, as far as possible, well-established scales in the existing literature, with appropriate modifications for the purpose of the measurement questions. Four types of variable scales are stated as follows:

(1) The Organizational Support scale. This study measured the support mainly using the Organizational Support Scale developed by Ling et al. (2006) and Tang (2018), which is found suitable for Chinese primary and junior high school teachers. The scale, which adopts the 5-point Likert scale, has been proven to have good reliability in numerous studies (Gan and Xu, 2018).
(2) The Job Crafting scale. The Slemp and Vella-Brodrick (2013) job crafting questionnaire was used, for example “I will use new skills to improve my work”. The 5-point Likert scale has undergone a rigorous process of translation and back-translation, and has been used by many other researchers with good reliability and validity (Liu et al., 2019).
(3) The basic psychological needs scale. The Chinese version of the Basic Psychological Needs Scale in use was proposed by Liu et al. (2013). This scale was revised, following a large number of sampling measurements based on the Gagne Basic Psychological Needs Scale (Gagne, 2003). The revised scale uses the 5-point Likert scale, and has been verified by researchers for reliability and validity (Zhang et al., 2017).
(4) The teacher well-being scale. The Organization for Economic Co-operation and Development [OECD] (2020) first included the Teacher Well-being Scale assessment in the PISA2021 mathematics framework, providing a holistic, systematic, universally suitable, and scientific conceptual framework and question items for measuring the teacher well-being. Following a rigorous translation and back-translation process, the scale was appropriately adapted and utilized as the teacher well-being scale for this study.

## Results

### Tests of reliability and validity

SPSS 26.0 was applied to examine the reliability and validity of the scales in this study. The Cronbach’s coefficient alpha values for all constructs were more significant than the benchmark value of 0.8 (see Table 2), and the deletion of any of the items might not significantly increase the alpha value, which indicates good internal consistency of the questions measured within a single dimension (Nunnally, 1978). The Cronbach’α value of the entire scale in this manuscript reads 0.954 suggesting a high overall reliability.

**TABLE 2:** Testing of reliability and convergent validity.

Variable	Cronbach-α	CR	AVE
Organizational support	0.951	0.953	0.742
Job crafting	0.914	0.916	0.647
Basic psychological needs	0.854	0.860	0.554
New math teachers’ well-being at primary and junior high schools	0.846	0.845	0.531

This study used more mature scales from home and abroad and conducted a pre-survey before the formal distribution of the questionnaire, with the question types revised repeatedly to ensure the accuracy of the questionnaire. This study explored both convergent validity and discriminant validity. Concurrent validity measures whether different items in a scale can reflect the same latent variable and is judged with two indicators: compositional reliability (CR) and average variance extracted (AVE). The judgment criterion, which had been proposed by Fornell and Larcker (1981), was put in use. At CR value of 0.7 or above, the consistency is measured higher, and the value is deemed better. And at an average variance extracted (AVE) of 0.5 or above, the variable has desirable convergent validity. Table 2 shows that the CR values in this study are more significant than the benchmark value of 0.7 and the AVE values more remarkable than the expected value of 0.5, which indicates that the scale was of satisfactory convergent validity.

Discriminant validity is the presence of a low correlation or significant difference between the potential trait represented by a variable and the trait represented by other variables, obtained by the square root of the AVE and the correlation between the other variables. In case of a greater square root of the AVE of a variable than the correlation coefficient between that variable and the other variables, discriminant validity between the variables is assessed as good. As shown in Table 3, the AVE open root values for each measure are greater than the correlation coefficients between that measure and the other ones. Therefore, the measurement model is readily of good discriminant validity.

**TABLE 3:** Discriminant validity testing.

Dimension	Organizational support	Job crafting	Basic psychological needs	New math teachers’ well-being at primary and junior high schools
Organizational support	**0.861**			
Job crafting	0.544**	**0.804**		
Basic psychological needs	0.679**	0.717**	**0.744**	
New math teachers’ well-being at primary and junior high schools	0.663**	0.570**	0.655**	**0.729**

The diagonal bold figure is the arithmetic square root of AVE. **P < 0.01.

### Relevant analysis of the variables

The mean value and standard deviation of all the variables in this study were calculated using SPSS 26.0 to work out the correlative coefficients between the variables and obtain the Pearson correlation coefficient matrix (the two-tailed test) for each variable analyzed, which is illustrated in Table 3. The results show the significant positive correlations among the variables of institutional support, occupational reshaping, basic psychological needs, and the teacher well-being.

### Model fit test

The model fit is the degree of consistency between the theoretical and sample models. The model goodness of fit can be examined using AMOS 24.0. A model fit is assessed as good when the figure is between 0 and 3. As the good fit index GFI, the adjusted AGFI, relative fit index TLI, and comparative fit index CFI exceed 0.9, the closer to 1.0 suggest a better goodness-of-fit between the data and the model, and the greater than 0.8, is an acceptable model. The model is assessed as a good fit given that the variability index RMSEA is less than 0.080 (Wu, 2000).

The testing results are shown Table 4, in which = 2.401, GFI = 0.837, AGFI = 0.800, TLI = 0.928, CFI = 0.928, and RMSEA = 0.076. Therefore, the sample model is affirmed to have better goodness of fit.

**TABLE 4:** Model fit indices.

Fit index	χ^2^/*df*	GFI	AGFI	TLI	CFI	RMSEA
Reference value	<3	>0.8	>0.8	>0.9	>0.9	<0.08
Examined value	2.401	0.837	0.800	0.919	0.928	0.075

### Path analysis and hypothesis testing

#### Direct effect testing

Four potential variables and their respective measure items were introduced into the set conceptual data model, and the study hypotheses proposed in the model were tested using the IBM SPSS AMOS 24.0 maximum likelihood estimating approach to examining whether the hypotheses would be supported by the parametric test structure. The final results are presented in Table 5, in which the standardized path coefficient of organizational support on the occupational well-being of new math teachers at primary and junior high schools, β = 0.218 (*t* = 4.870, *P* < 0.001), indicates a significant positive effect of organizational support on the teacher well-being, by which hypothesis H1 was verified; the standardized path coefficient of job crafting on the aforesaid new math teacher well-being, β = 0.135 (*t* = 2.023, *P* < 0.05), a significant positive effect of job crafting on the teacher well-being, by which the hypothesis H2 was verified; the standardized path coefficient of basic psychological needs on the teacher well-being, β = 0.209 (*t* = 2.530, *P* < 0.01), a significant positive effect of basic psychological needs on the teacher well-being, by which the hypothesis H3 was verified; the standardized path coefficient of institutional support on basic psychological needs, β = 0.343 (*t* = 7.356, *P* < 0.001), a significant positive influence of organizational support on basic psychological needs, by which the hypothesis H4a was tested; and the standardized path coefficient of job crafting on basic psychological needs, β = 0.562 (*t* = 7.645, *P* < 0.001), a significant positive effect of job crafting on basic psychological needs, by which the hypothesis H5a was verified.

**TABLE 5:** Path correlation testing.

Hypothesis	Path	Standardized coefficient	Standard error	*t*-value	Significance	Hypothesis tested
H1	Organizational support ⇢ The teacher well-being	0.218	0.045	4.870	***	YES
H2	Job crafting ⇢ The teacher well-being	0.135	0.67	2.023	0.043	YES
H3	Basic psychological needs ⇢ The teacher well-being	0.209	0.083	2.530	0.011	YES
H4a	Organizational support ⇢ Basic psychological needs	0.343	0.047	7.356	***	YES
H5a	Job crafting ⇢ Basic psychological needs	0.562	0.074	7.645	***	YES

***P < 0.001.

#### Intermediary effect testing

Based on a simple correlation analysis of the variables, this study used the structural equation modeling to provide insight into the correlation between the variables and thus to examine the intermediary role of basic psychological needs. Bootstrapping was used to sample 5,000 times and 95% confidence intervals were set to test the significance of the mediating effect of organizational support, job crafting and the teacher well-being. Mackinnon et al. (2004) concluded, through experiments, that the bias-corrected method, a non-parametric Bootstrap approach, was optimal. Therefore, only the bias-corrected confidence intervals are reported in this study. With reference to the results of the mediating effects test (see Table 6), the intermediary path effect of the organizational support to the occupational well-being of new math teachers at primary and junior high schools was 0.138. So the mediating effect of basic psychological needs was significant (none of the confidence intervals contain 0, *P* < 0.05), and it can be assumed that hypothesis H4b is valid; and the intermediary path effect value of job crafting on the teacher well-being was 0.167, which indicates that the mediating effect of basic psychological needs was significantly present (None of the confidence intervals contain 0, *P* < 0.05), and so hypothesis H5b holds. To sum up, basic psychological needs have a mediating effect in promotion of the teacher well-being with organizational support and job crafting.

**TABLE 6:** Intermediary effect testing.

Path	Effect value	95% confidence intervals	*P*
Organizational support ⇢ Basic psychological needs ⇢ The teacher well-being	0.138	[0.11, 0.311]	0.035
Job crafting ⇢ Basic psychological needs ⇢ The teacher well-being	0.167	[0.05, 0.382]	0.046

**FIGURE 2:**
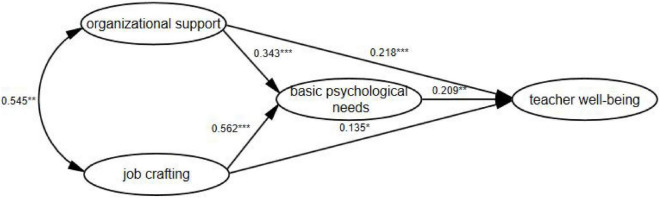
The path relationships between the variables of organizational support, job crafting, basic psychological needs, and teachers well-being. *P < 0.05, **P < 0.01, ***P < 0.001.

After the above path analysis and hypothesis testing, all the influential paths of the model met the criteria of statistical significance. The specific path relationships between the variables of organizational support, job crafting, basic psychological needs and teachers well-being in the research model are shown in Figure 2.

## Discussion

Compared with prior research findings on the teacher well-being, this study find that in the following three facets.

Firstly, researchers have ever applied the self-determination theory to explain the formation mechanism of occupational well-being (Tims and Bakker, 2010; Yen, 2012; Slemp and Vella-Brodrick, 2013), but there are some overlapping influencing factors on the occupational well-being, such as the interaction with both organizational support and job crafting (Zou et al., 2015). Therefore, this study has found that organizational support and job crafting have a direct positive effect on the occupational well-being of new math teachers at primary and junior high schools, which happens to be consistent with some other researchers’ views (Wang and Xu, 2008; Wei et al., 2018). Unlike the previous studies, the study has also found that organizational support and job crafting also indirectly affect teacher well-being through a survey of basic psychological needs. At the same time, this study intended to explore the theory with a multi-level dynamic formation mechanism model of occupational well-being. That may help deepen the understanding of the factors influencing institutional backing and occupational reshaping and thus extend the research on the factors contributing to teacher well-being.

Secondly, Current research on the factors influencing the teacher well-being has focused on work stress (Yang, 2014), psychological capital (Ning, 2017), and job autonomy (Zeng et al., 2021), still leaving room for further studies on the mechanisms influencing the well-being (Wang, 2019). This study found that organizational support, job crafting, and basic psychological needs may contribute to the occupational well-being of new math teachers in primary and junior high schools, with the basic psychological needs mediating effect. These findings could fill the gap in the studies on the factors influencing the occupational well-being of primary and junior high school teachers (Wang, 2019), enriching those on the antecedent variables of the teacher well-being, and eventually, advances on the role of the mechanisms influencing the well-being.

Thirdly, this study also takes into account such factors as the teaching experience of the subjects (Hu and Zhou, 2014). The teacher well-being varies across teaching years and influences different factors (Ling et al., 2016). Besides, it may also change from country to country, in different cultural backgrounds, even in the similar context within China, the teachers may have different levels of well-being (Peng and Yin, 2021). Nonetheless, quite few studies of the teacher well-being have been made with new teachers at primary and junior high schools (Pei and Li, 2015). Moreover, there are fewer studies on teachers instructing a particular subject at primary and junior high schools (McCallum et al., 2017). Therefore, this study investigates the teacher well-being of new math teachers at primary and junior high schools in China to further study teacher well-being.

## Conclusion

While applying the theory of multi-level dynamic formation mechanism model of the teacher well-being, this study has conducted an empirical investigation by the structural equation modeling with 248 samples as new math teachers at primary and junior high schools. A preliminary model has been constructed for the roles of organizational support and job crafting in affecting the occupational well-being of the new math teachers at the schools. To this end, the study has found that the correlative roles among the organizational support, job crafting, basic psychological needs and occupational well-being with primary and junior high school teachers, and that there exists a mediating effect of basic psychological needs with organizational support, job crafting, and the teacher well-being.

Concerning practical implications, this study illustrates that organizational support has a significant positive effect on the occupational well-being of the new math teachers at the schools. These teachers’ perceptions of the school policies and management, resource support, and humanistic care determine whether they are able to achieve their psychological satisfaction. Furthermore, as their reasonable requests are met and assistance offered promptly upon encountering any problem, the teacher well-being will also be enhanced with higher satisfaction with their school. Therefore, schools should establish appropriate policies to support their teaching and living. For example, schools should understand and help new math teachers to deal with the problems they encounter at work, create a positive working atmosphere for them, and communicate with them before making decisions related to their work; and offer guidance and feedback on their work where necessary. In other words, schools should fully understand the importance of new math teachers’ well-being to their schools and practice the well-being specific policies from the perspective of institutional support.

Additionally, as shown in the study, job crafting significantly positively affected the occupational well-being of new math teachers at primary and junior high schools. Also, to meet their needs, based on their own needs and job characteristics, new math teachers can adopt proactive behaviors, to achieve a sustainable person-job fit. They also actively construct their job crafting by re-examining the connections to their tasks, work environment, abilities, and interests and transforming their work. Meanwhile, the schools are advised to develop appropriate job crafting intervention programs considering the specific circumstances of new math teachers to enhance their reshaping capacity. Besides the findings of this study, the schools may also leave some room for bottom-up job crafting by new math teachers, encourage them to exert their initiative, and thus enhance their occupational well-being.

This study remains limitations in several aspects. First of all, the subjectivity of measurement indicators is hardly avoidable. Although its design may reduce bias and errors to a certain extent, this study adopted domestic and international scales and conducted a pilot study before the formal investigation to minimize the impact of subjective errors. However, such errors may still inevitably exist, which will be further minimized in future studies through in-depth interviews based on the Grounded theory and qualitative survey. Secondly, there are quite a few influencing factors on the occupational well-being of new math teachers at primary and junior high schools. This study just investigated three factors, still losing sight of many others, which can be further studied in later studies by introducing possibly sufficient variables from more diverse perspectives. Thirdly, the sample size of this study was only confined to new math teachers at primary and junior high schools in some provinces, which should be extended across the country in China, with a much larger size of samples and rigorously verified error-free data. Additionally, primary and junior high schools are two different contexts. This study does not consider the differences between teachers in further education segments due to time and effort constraints. In future studies, we can consider the differences in the relationship path relationships between the variables of institutional backing, job crafting, basic psychological needs, and teacher well-being. Therefore, the author hopes that future studies on the occupational well-being of new math teachers at primary and junior schools will be further optimized, in both diversity and magnitude.

## Data availability statement

The raw data supporting the conclusions of this article will be made available by the authors, without undue reservation.

## Author contributions

LW and FP conducted the data analyses, interpreted the results, and drafted the manuscript. NS provided feedback and co-wrote the final submission. LW, FP, and NS conceived the idea of the study, designed the study, contributed to the manuscript revision, and approved the submitted version.
